# Hidden suicides: focus on England and Wales – comparison with other nations

**DOI:** 10.1192/bjp.2025.20

**Published:** 2025-08

**Authors:** John Snowdon

**Affiliations:** 1 Sydney University, Sydney, Australia; 2 Department of Psychiatry, Concord Hospital, Sydney, Australia

**Keywords:** Accidental death, misclassification, psychological autopsy, suicide rates, undetermined deaths

## Abstract

Most deaths around the world are certified, registered and then ‘coded’ for statistical purposes. Misclassified (‘hidden’) suicides are deaths assigned an ICD code that is either erroneous or that should never be specified as a cause of death. Public health strategies depend on provision of accurate mortality statistics. Suicides are under-counted, largely through misattribution to natural disease, accident, ill-defined or unknown cause (code R99) or an event of undetermined intent. Proportions of suicides misclassified to each of these codes vary between nations. It is recommended that psychological or verbal autopsies be used when investigating external deaths of uncertain cause or intention, and some R99 deaths. This applies in Britain and wherever unusual patterns of deaths could signal hidden suicides – exemplified by high rates of drug deaths in North America.

Suicides are under-counted in most (maybe all) nations. Jurisdictions vary in the extent to which deaths of people who killed themselves are being assigned ICD codes that misclassify the cause or manner of death. Misclassified suicides have been called ‘*hidden suicides*’.^1–3^

## Mortality statistics: how accurate are they?

The number of suicides per year in the world is unknown. Undoubtedly, some people who intentionally kill themselves take steps to ensure their deaths are not recognised as self-inflicted. In other cases, those who report that a person has died, and those called to certify a death, may purposely hide the fact that a death was probably by suicide. This appears to be more common in certain nations than in others, for various reasons – for example, where there are social, cultural or religious reasons for suicide being stigmatised, or where suicide is still a crime, or because insurance companies can refuse payments in cases of suicide. In such situations, the death might well be certified as owing to natural causes, especially if the decedent is elderly and known to have suffered medical problems. In many countries, suicide rates peak in late old age, and it seems plausible that rates of unacknowledged suicide also reach peaks in late life. People ending their lives to escape age-associated infirmities or problems could well be among those wanting others not to know their deaths were suicides; physicians may be reluctant to request autopsies on their older patients, even if suspicious about what caused their deaths.

Most of the 194 nation members of the World Health Organization (WHO) provide mortality statistics to the WHO Mortality Database^4^ annually. However, from more than half the nations, the quality of the reported statistics is poor or, at best, moderate.^5,6^ This has been attributed, largely, to under-resourcing, under-availability of physicians to certify deaths and/or inadequate skills, training and organisation of administrative officers tasked with responsibility for gathering relevant information about deaths in their nation. To compare mortality statistics around the world (for public health and other purposes), nations are required to use the ICD when coding diseases and causes of death. Unfortunately, classification problems have arisen following inclusion in later editions of the ICD of various codes that should never be assigned as the underlying *causes* of death, such as ‘senility’ (R54), ‘cardiac arrest’ (I46) and ‘heart failure’ (I50). They have been termed ‘garbage codes’.^7^

Education and more adequate resourcing have resulted in improvements in quality where it was needed,^6^ but still there is excessive use of garbage codes rather than ‘usable’ codes. Some nations have provided mortality figures showing that half or more of their deaths had been assigned ‘undetermined cause’ codes. Responding to recognition that ‘unusable’ (garbage) coding has resulted in unacceptable degrees of misclassification of suicides, and to improve accuracy regarding the global suicide rate, WHO has facilitated *reassignment* of ‘unusable’ codes to what have been deemed plausible ICD alternatives, using algorithms developed by Global Burden of Disease (GBD) study researchers.^8^ As a result, estimated suicide rates in some nations, as documented by WHO, have been more than twice the rates reported to WHO’s database by those countries. A GBD study in India estimated there had been 230 314 suicides in 2016, in contrast to an official 2015 report of 133 623 suicides.^9^ The number of worldwide suicides in 2016 was estimated as 817 000.^8^

Although reassignments have led to documentation of a higher and more realistic estimate of the global suicide rate, many suicides are believed to remain unrecognised and under-counted. Among these, and added to those mentioned above, is the fact that some countries do not provide sufficient resources to assiduously investigate all deaths where the cause is uncertain. This is understandable in lower income nations. However, being rich or being assessed as providing mortality data of high quality have not necessarily ensured accuracy of suicide data.^10^ Even in high-income countries, forensic autopsy appears to be used uncommonly when investigating the cause of death.^11,12^

One way of reducing data inaccuracies is to add ‘psychological autopsy’ or ‘verbal autopsy’ interviews as part of investigative processes. Psychological autopsy has been described as ‘an intensive retrospective analysis of the decedent’s mental state and intentionality based on semi-structured interviews with potential key informants’.^13^ Although initially used by police and psychologists in investigating unexplained deaths, in recent years psychological autopsies have become more often utilised as a research tool for investigating causative factors in relation to suicide. Rockett et al^14^ recommended ‘a reversal of the now common practice of using psychological autopsies to enrich the understanding of validated suicides only, at the expense of helping resolve intentionality in deaths from equivocal, ill-defined or unknown causes’. They suggested that if findings from psychological autopsies (including following drug overdose fatalities) were more available to coroners and medical examiners in nations that have them, this would improve accuracy in estimating true suicide rates. There is good reason to request psychological autopsy more often, although cost considerations are likely to limit its use.

Mortality data have been obtained using verbal autopsy in various countries that do not have fully developed civil registration and vital statistics systems. Routine, validated, automated verbal autopsy methods have been recommended, particularly in relation to non-hospital deaths.^6^ Verbal autopsy interviews of people close to the decedent are conducted by trained non-medical community workers and focus (like psychological autopsies) on health history and circumstances before the death. The range of gathered data and accuracy in identifying cause of death varies between jurisdictions, although a standardised verbal autopsy format is available from the WHO. Verbal autopsy is, in effect, a simplified version of ‘psychological autopsy’. In Indonesia, Onie et al^15^ emphasised the importance of recruiting ‘local’ people to conduct verbal autopsy interviews, and for them to assure confidentiality when seeking details about deaths in their districts. Their verbal autopsy data enabled development of Indonesia’s first suicide statistics profile, although their study revealed a lower suicide rate than that estimated by GBD researchers.

## Hidden suicides

Rates of suicide coded, knowingly or unknowingly, to natural disease categories are unknown. Other ICD-10 codes thought to most commonly ‘hide’ suicides^3^ are as follows:

- event of undetermined intent (EUI) (Y10–34, listed as garbage codes);
- accidental death caused by poisoning (X40–49), drowning (W65–74), fall from a height, traffic or rail injury or other injury, including X59 (unspecified; a garbage code);
- ill-defined or unknown cause deaths (R99, a garbage code).

The codes assigned to accidental and EUI deaths all denote an *external* cause of morbidity or mortality. Contrastingly, by definition, the code R99 is assigned to deaths for which classifiers can find no evidence to allow a decision about whether the causative condition was an external injury or a natural or communicable disease (and if so, which one).

## Event of undetermined intent death rates in the UK

Since the early 2000s, the Office of National Statistics (ONS) in England and Wales, which gathers mortality data from the whole of the UK, has combined numbers of deaths coded X60–84 (suicide) and Y10–34 (EUI) when publishing *official* UK suicide statistics. Research in England by groups of psychiatrists (including Linsley et al^16^) showed that rate patterns of deaths judged by coroners to be suicides were similar to those of decedents assigned an undetermined/open verdict (one where there was reasonable doubt about whether the person intended to die). There was a significant difference in social class between open and suicide decedents, but their psychiatric histories were similar. The open verdict group were more likely to have died from drowning or a fall from a height, and were less likely to have left a suicide note. The ONS supported the psychiatrists’ conclusion that most undetermined (EUI) deaths were hidden suicides, and introduced a definition of suicide that embraced ‘deaths with an underlying cause of intentional self-harm (ages 10 years and over) and deaths with an underlying cause of event of undetermined intent (ages 15 years and over)’.^17^

The ONS, like statistics offices in other nations, reports X60–84 and EUI (Y10–34) mortality rates separately to WHO’s Mortality Database.^4^ In general, nations have not adopted the UK policy of counting all EUI deaths as suicides. In this article, population rates are reported per 100 000: the *official* suicide rate in England and Wales, having averaged 10.5 in 2000–2005, was 9.15 in 2013–2022. It fell to 8.39 in 2017, was 9.55 in 2022, and in 2023 was 9.97 (*n* = 6069); the latter figure is likely to be revised upwards in the next 2 years as data from coroners’ offices become available. The rate of deaths coded X60–84 averaged 7.61 in 2013–2022, rising from 7.14 to 8.50; it was 9.11 in 2023. The EUI death rate averaged 1.54, falling from 2.10 in 2013 to 1.47 in 2019, and between 0.99 and 1.05 in 2020–2023. Similar falls have been reported in other nations (see below). Increases in X60–84 rates with corresponding falls in Y10–34 rates in the UK have followed a change in British legislation: since mid-2018 the civil code (balance of probabilities) has been used rather than the criminal code (beyond reasonable doubt) when deciding whether a death was a suicide. Because in England and Wales numbers of X60–84 and Y10–34 deaths are added to calculate official suicide rates, the change in legislation has led to a relatively small change in what is *officially* reported as the suicide rate.

**Fig. 1:**
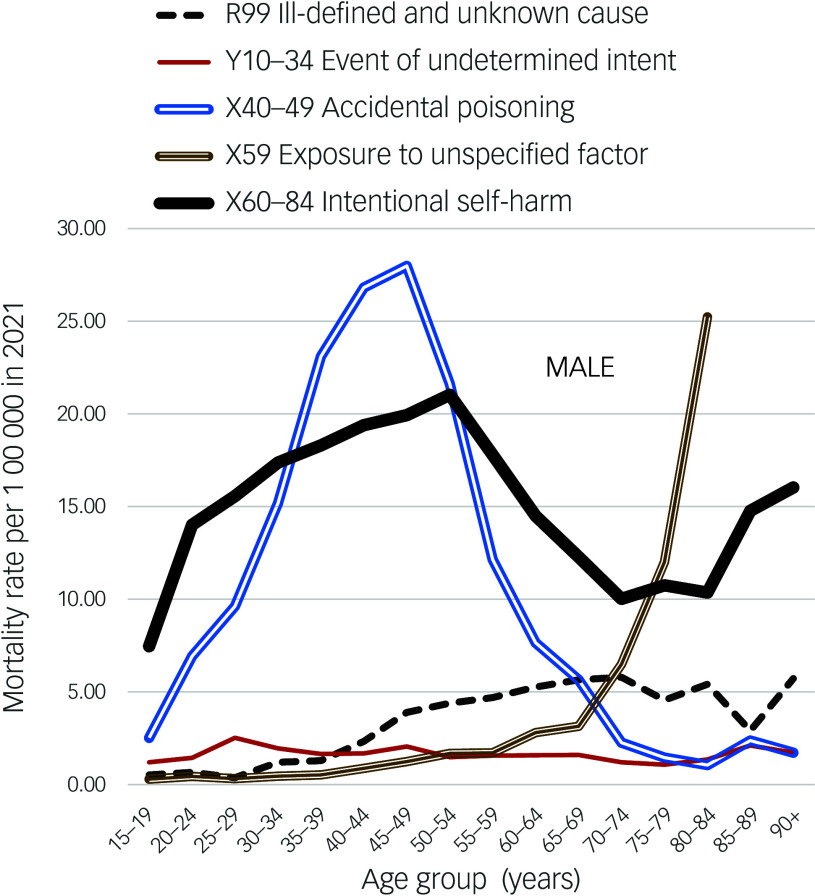
Male mortality rates in England and Wales, 2021.

**Fig. 2:**
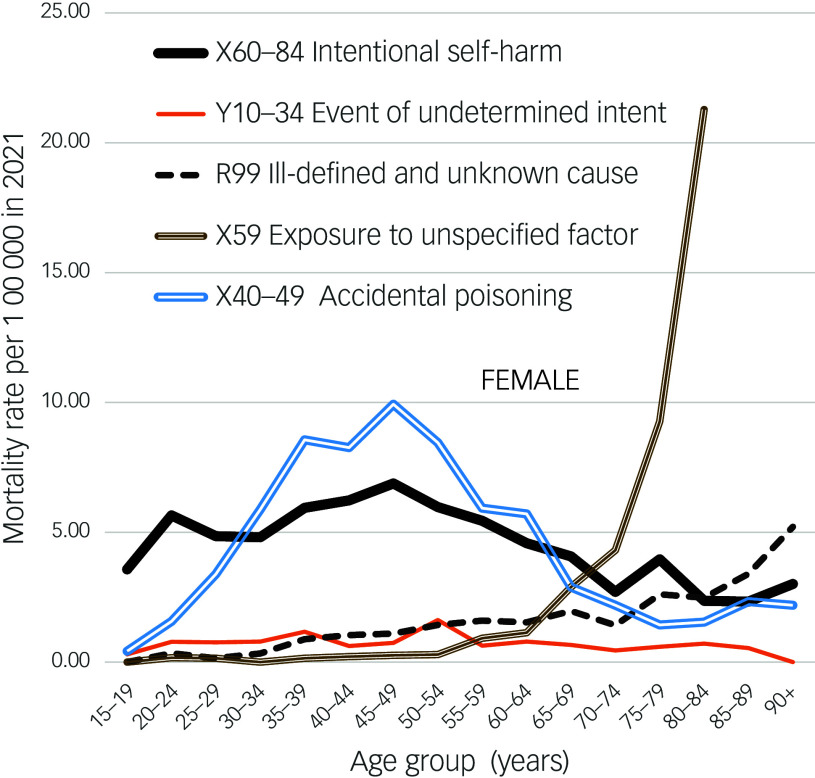
Female mortality rates in England and Wales, 2021.

Male and female age patterns of suicide in England and Wales in 2021, and of deaths in 2021 assigned to codes that may ‘hide’ suicides, were graphed. Figures 1 and 2 show relatively unchanging patterns of EUI rates across the age range, and relatively small increases in R99 rates in late life when compared to most other nations. The finding that the figures show notable differences between age patterns of suicide and EUI deaths, as well as the halving of EUI rates, provide reasons for re-examining what percentage of UK cases currently being coded as EUI deaths were probable suicides.

## Event of undetermined intent death rates in other nations

EUI deaths have been declared the mortality category most prone to contain misclassified suicides^18,19^; rates in selected populous nations that have been assessed as providing high-quality mortality statistics to WHO have been compared.^20^ Portugal and Korea reported rates of 7.6 and 3.5, respectively, but further investigation is needed to determine what percentages of these EUI deaths were ‘hidden suicides’. In Sweden, 70–75% of EUI deaths were later reclassified as suicides.^21^ A study in Israel,^22^ comparable to Linsley et al’s^16^ in England, showed that 10.6% of deaths assigned to an EUI code were probably suicides. Between 2015 and 2021, Canada’s EUI death rate fell from 2.2 to 0.8. Rates in Japan, the USA, Germany and France have ranged between 1.0 and 2.0. Australia’s fluctuated between 0.8 and 0.5 in 2013–2022. Rates in Chile, Italy, Spain and the Netherlands have recently been 0, 0, 0.1 and 0.2, respectively. Clearly, in these latter countries, misclassification of suicides as EUI deaths is uncommon or non-existent.

Quality of mortality statistics in Russia and various neighbouring countries is relatively low; their suicide and EUI death rates are high, the latter being largely attributed to homicide. In the early 2000s, rates in Russia were 28.0 (EUI), 29.1 (suicide) and 20.8 (homicide).^23^

As well as remarkable differences in EUI death rates between nations, there have been marked differences in age patterns. Graphs of Korea’s age patterns of EUI rates *and* of suicide across adulthood showed exponentially increasing rates, with the male EUI rate reaching 84.0 at age 85 years or more.^24^ Mexico and various other nations recorded unimodal (convex) male EUI death patterns, but (in contrast to Korea) Mexico’s suicide patterns (both genders) were downward-sloping from peaks in young adulthood. Germany’s age pattern was relatively flat; the comparable suicide pattern was upward-sloping with a steeper increase in late life.

**Fig. 3:**
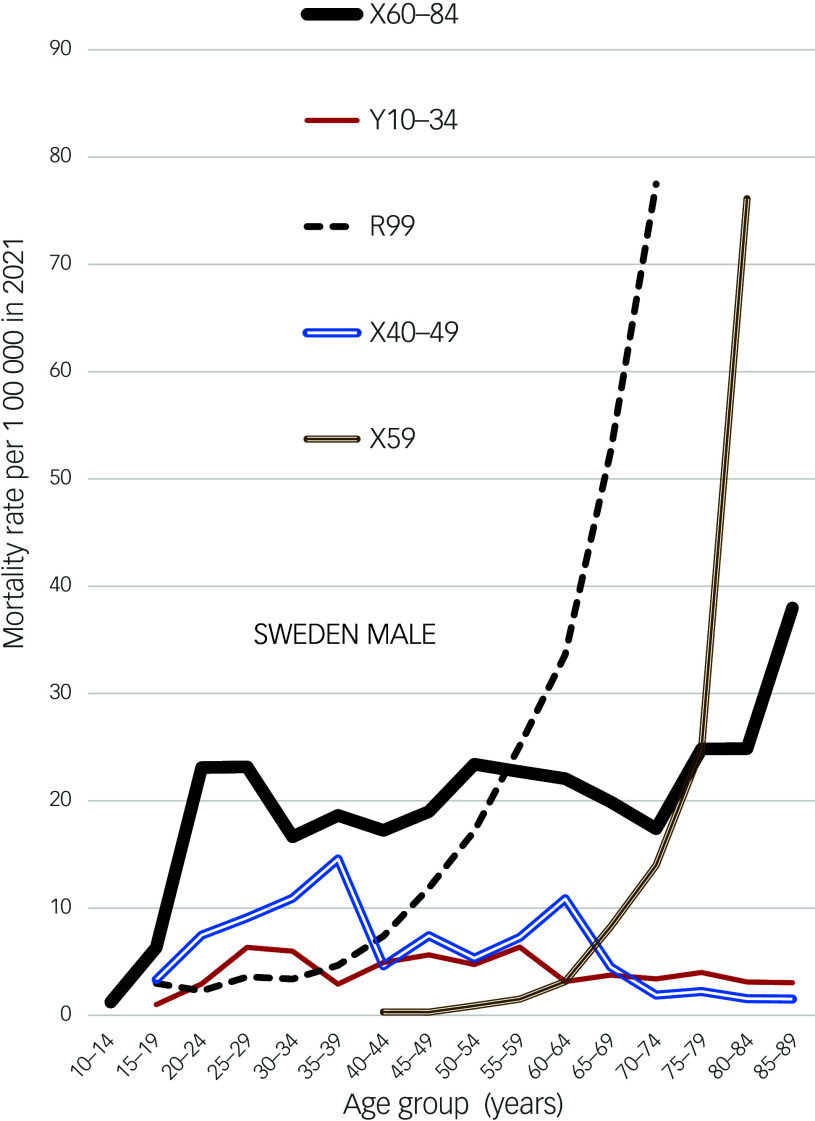
Male mortality rates in Sweden, 2021.

In contrast to these and British EUI age patterns, corresponding patterns in the USA and Australia have been bimodal, with highest peaks in early middle age and second peaks in later life. EUI and suicide age patterns somewhat resembled each other. In Sweden the male age patterns of EUI deaths and suicide were similar up to age 70–74 (the suicide rate being four times higher), but the Swedish suicide rate increased sharply in late old age while the EUI death rate did not (Fig. 3).

In all these nations, except in those where rates are at or close to zero, EUI death rates of females were lower than those of males, but age patterns of the genders commonly resembled each other: peak Korean, Mexican, Argentinian and German male and female EUI death rates were in late life, as was the Japanese male rate; the Japanese, UK, US and Australian female EUI death rates peaked in middle age.

There are disparities between the medico-legal investigative resources and procedures of different nations and populations regarding verdicts pronounced in cases of undetermined deaths. It is reasonable to ask to what extent nations with low EUI death rates have policies and processes in place to discourage coders and death certifiers from assigning Y10–34 codes. Across the USA, the percentage of drug intoxication deaths classified as undetermined (EUI) varies greatly between coroners and medical examiners, with one report quoting a range from 1% to 85%, with an average of 8%.^25^

Similarities between age patterns of suicide and EUI death rates in some nations support opinions that in those nations EUI deaths were probably suicides. In others, such as Mexico, the patterns were very different. If this means they were probably *not* suicides, there need to be studies (for example, using verbal autopsies) of whether, with more intensive investigation, they can be identified as accidental deaths or homicides.

## Accidental deaths

There is evidence that, at times, various nations have encouraged those who code or certify deaths to assign an accidental death code in cases where there is uncertainty about whether a death was a suicide. In England, around 1990–2005, the proportion of coroners’ verdicts of accidental death (particularly by poisoning) doubled and there was a corresponding reduction in suicide verdicts.^26^ In both the USA and Canada there is evidence of under-counting of drug suicides caused by misclassification as accidental drug overdose deaths.^25,27,28^ Misclassifications among poisoning deaths may have led to underestimation of suicide rates in the USA by 10–30%.^29^ There should be increased psychological autopsy/verbal autopsy use in cases of drug overdose fatalities in countries reporting high rates.

Commonly there is doubt, in cases of drowning, about whether the death was a suicide.^2,30^ In the absence of a suicide note or other evidence that the decedent was depressed or conflicted and considering death as a solution, an EUI or accident code may be assigned. The same may apply in cases of single-occupant car crashes, falls from a height and other fatal injuries. In such cases, an expertly conducted psychological autopsy or verbal autopsy is desirable. Whether they are assertively and assiduously investigated not only depends partly on whether skilled resources are available and affordable, but also on training, commitment and attitudes of those given responsibility for gathering relevant information.

X59 has been described as a garbage code assigned when death was deemed as caused by ‘accidental exposure to an unspecified factor’. When Norwegian deaths coded X59 were redistributed to external cause groups, 97.4% were attributed to accidental falls and 1.7% as suicides. Most X59 deaths in Norway^31^ and Sweden (Fig. 3) were of people aged 70 years or more. This is consistent with age patterns of X59 rates in England and Wales, where recently the X59 rate was reported as 3.4: the proportion of UK X59 deaths that were hidden suicides is likely to have been small.

## Ill-defined and unknown cause (R99) deaths

While intimating that many deaths coded as accidental poisonings were probably misclassified suicides (see above), Rockett et al^14^ asserted that ‘the two most imprecise cause-of-death categories that we documented as being highly prone to contain misclassified suicides [are] injury of undetermined intent and ill-defined and unknown causes’. They commented that R99 is a less precise category than Y10–34 (EUI), since it fails to reveal whether the death was from injury or disease. It should be noted, however, that Y34 (unspecified event, undetermined intent) is also imprecise.

**Fig. 4:**
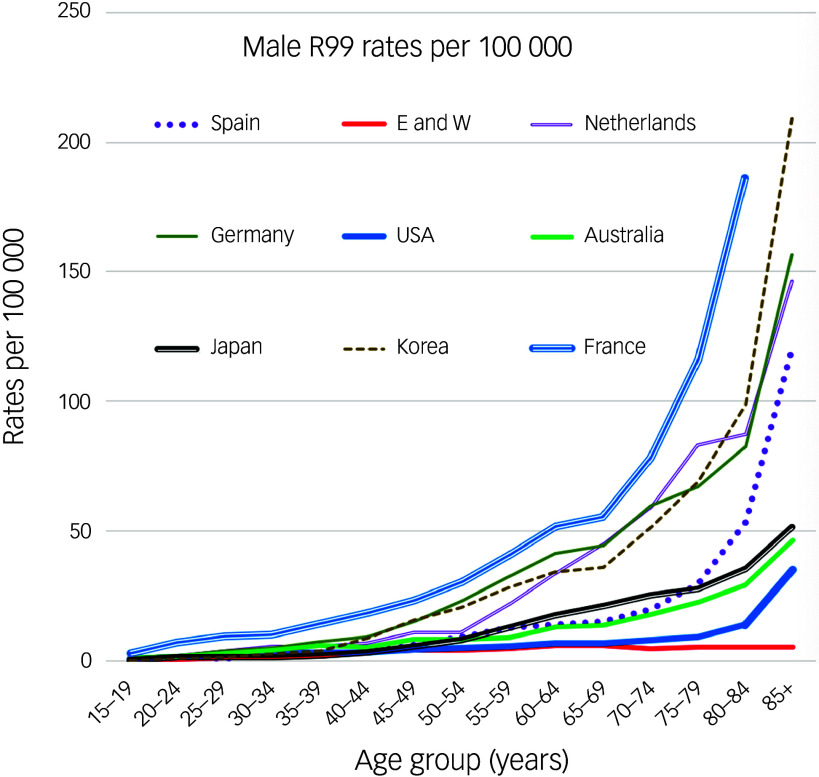
Male R99 rates in selected nations in 2015. E and W, England and Wales.

There is good reason to believe that most R99 deaths are not suicides. Figure 4 shows why. The fact that in most nations graphs of the R99 rates rise exponentially across late life is likely to be largely because it can be more difficult to decide what was the underlying cause of death when an older person dies. Multimorbidity is common. At the end of life their physicians may not have wanted to inflict investigations on a person who seemed close to death. In the Tel Aviv study mentioned above, 53 (1.9%) of the deaths coded as ‘unknown causes’ were believed to be probable suicides, and the authors concluded that ‘unknown causes’ are a prime contender for containing misclassified suicides.^22^ However, their 53 cases were all known to have had ‘external’ causes and perhaps should have been coded as EUI deaths. No other studies (and in particular, no psychological autopsy studies) of series of R99 deaths have documented the percentage of these deaths that were probably suicides.

R99 death rates (male and female separately) in most nations around the world are published annually. The mean rate in England and Wales in 2013–2022 was 2.10 (male 3.19, female 1.30). These rates are among the lowest recorded from different nations (the rate in Greece is lower). Equally striking is the difference between graphs of the *age patterns* of R99 death rates in England and Wales and those of other nations. Most climb exponentially in late life (as in Fig. 4), whereas the age patterns in England and Wales slope gently upwards to 5.07 (male) and 2.92 (female) at age 80–84 years, and 5.38 (male) and 5.92 (female) at age 85+ years (Figs. 1 and 2). Rates across the age range to 70 years are even lower in Mexico, but then rise. The male rates at age 80–84 years in the same year in the USA, Australia, Mexico, Japan, Spain and Korea were, respectively, 13.74, 28.85, 31.43, 35.6, 53.09 and 98.07. At 85+ years they were much higher. Reasons why England and Wales have relatively low R99 rates across all age groups (including late life) compared to most other nations are worthy of study.

The R99 code is commonly documented as a ‘place-keeper’ while waiting for coronial verdicts to be pronounced. This is why, when countries such as England and Wales and Australia first publish a year’s mortality statistics they show higher R99 rates and lower suicide rates than those published when they release revised reports a year or two later.^32^ Deaths initially coded R99 but later judged as suicides were in effect ‘hidden suicides’ until finalisation of the cases. Those studying the statistics need to note whether they are examining finalised data before coming to final conclusions about rates of suicide, R99 deaths, accidents, EUI deaths or, indeed, any ICD-coded condition in a particular year. Some countries never revise mortality statistics after their first publication, even if further information about deaths becomes available; various nations progressively update their mortality figures. Others publish finalised statistics only once, but not until some years after the year the statistics relate to, and therefore there is little need to update.

The quality of a country’s mortality statistics is partly judged by its R99 rates. Because in most nations deaths in late life coded R99 probably were largely attributable to physical illnesses, with proportionally few coded as suicides, psychological autopsies relating to deaths coded R99 among people of advanced age are likely to reveal few suicides; even if deaths that appeared to be obviously from natural causes were excluded, studies of a consecutive series of R99 cases would be expensive. On the other hand, if middle age suicide rates are low in a country that boasts suspiciously low accident and EUI rates in that age group, psychological autopsy studies could show whether the hidden suicide rate (coded R99) is high in middle age.

Suicides are under-counted. Primarily, this is because they are being misclassified to other ICD codes. Globally, misclassification of suicides as EUI, R99 and accidental deaths could exceed 30%, but rates of misclassification to one or other of the different categories vary in extent and direction (higher/lower) between nations. Different researchers have implied that EUI, R99 or accidental deaths can be ‘blamed’ to a greater extent than the others.

Within each nation, given adequate resources and investigations, and scrupulously comprehensive gathering of mortality data, using psychological autopsy or verbal autopsy in representative numbers of cases would allow more accurate estimates of suicide and each type of hidden suicide in the population. Poorer nations will need financial support for such work. Closer examination of British figures is needed.
